# Elucidating the multichromosomal structure within the *Brasenia schreberi* mitochondrial genome through assembly and analysis

**DOI:** 10.1186/s12864-024-10331-0

**Published:** 2024-04-29

**Authors:** Yuanyu Shan, Jingling Li, Xinmei Duan, Xue Zhang, Jie Yu

**Affiliations:** 1 College of Horticulture and Landscape Architecture, Southwest University, Chongqing, 400716 China; 2 Key Laboratory of Agricultural Biosafety and Green Production of Upper Yangtze River (Ministry of Education), Southwest University, Chongqing, 400715 China

**Keywords:** *Brasenia schreberi*, Mitogenome, Repeat analysis, MTPT, RNA editing, Phylogenetic analysis

## Abstract

*Brasenia schreberi*, a plant species traditionally utilized in Chinese medicine and cuisine, represents an early evolutionary stage among flowering plants (angiosperms). While the plastid genome of this species has been published, its mitochondrial genome (mitogenome) has not been extensively explored, with a notable absence of thorough comparative analyses of its organellar genomes. In our study, we had assembled the entire mitogenome of *B. schreberi* utilizing the sequencing data derived from both Illumina platform and Oxford Nanopore. The *B. schreberi* mitogenome mostly exists as six circular DNA molecules, with the largest being 628,257 base pairs (bp) and the smallest 110,220 bp, amounting to 1.49 megabases (Mb). Then we annotated the mitogenome of *B. schreberi.* The mitogenome encompasses a total of 71 genes: 40 of these are coding proteins genes (PCGs), 28 are genes for transfer RNA (tRNA), and the remaining 3 are genes for ribosomal RNA (rRNA). In the analysis of codon usage, we noted a unique codon preference specific to each amino acid. The most commonly used codons exhibited an average RSCU of 1.36, indicating a noticeable bias in codon selection. In the repeat sequence analysis, a total of 553 simple sequence repeats (SSRs) were identified, 1,822 dispersed repeats (comprising 1,015 forward and 807 palindromic repeats), and 608 long terminal repeats (LTRs). Additionally, in the analysis of homologous sequences between organelle genomes, we detected 38 homologous sequences derived from the plastid genome, each exceeding 500 bp, within the *B. schreberi* mitochondrial genome. Notably, ten tRNA genes (*trnC-GCA*, *trnM-CAU, trnI-CAU, trnQ-UUG, trnN-GUU, trnT-GGU, trnW-CCA*, *trnA-UGC*, *trnI-GAU*, and *trnV-GAC*) appear to have been completely transferred from the chloroplast to the mitogenome. Utilizing the Deepred-mt to predict the RNA editing sites in the mitogenome, we have identified 675 high-quality RNA editing sites in the 40 mitochondrial PCGs. In the final stage of our study, we performed an analysis of colinearity and inferred the phylogenetic relationship of *B. schreberi* with other angiosperms, utilizing the mitochondrial PCGs as a basis. The results showed that the non-coding regions of the *B. schreberi* mitogenome are characterized by an abundance of repetitive sequences and exogenous sequences, and *B. schreberi* is more closely related with *Euryale ferox*.

**Supplementary Information:**

The online version contains supplementary material available at 10.1186/s12864-024-10331-0.

## Introduction

*Brasenia schreberi* J.F. Gmel, a member of the family Cabombaceae within the order Nymphaeales, is a perennial aquatic flora. This archetypal angiosperm predominantly proliferates in regions including China, Japan, North America, and eastern Oceania [1, 2], and the wild species has been classified as a primary-grade key protected species in China [3]. *B. schreberi*, historically significant in traditional Chinese medicine and cuisine, is characterized by a rich composition of polysaccharides, proteins, polyphenols, trace elements, vitamins, and other biologically active compounds [4]. Medically, it exhibits antibacterial, anti-inflammatory, anti-tumorigenic, and hypoglycemic properties [5, 6]. Agriculturally, it has a longstanding history of cultivation in China, particularly in marshes and ponds, with a widespread distribution across southern provinces such as Hubei, Sichuan, Zhejiang, and Jiangxi. Currently, *B. schreberi* cultivation is concentrated in four primary production regions in China: Shizhu, Lichuan, Leibo, and Xihu. The economic significance of *B. schreberi* is mainly attributed to its young leaves, enveloped in a polysaccharide gel, which forms the main edible component [7]. The quantity and quality of this polysaccharide gel are directly influential in determining the plant's economic and nutritional value. Contemporary research on *B. schreberi* at the molecular level is limited. The focus is mainly on developing and applying various molecular markers. Notably, one study have employed RAPD and AFLP markers to investigate the conservation genetics of the species [1]. In another separate study [8], 28 SSR markers were identified in another separate study, leading to a subsequent investigation into the population structure of 21 *B. schreberi* populations across China using these markers.

Mitochondria in plants play a pivotal role in various biological processes including respiration, metabolism, programmed cell death, and male sterility, thus making them essential for the plant growth [9, 10]. Based on the endosymbiotic theory, plant mitochondria originated from an alpha-proteobacterium that was engulfed and formed a symbiotic relationship with eukaryotic host cells [11]. The mitochondrial genome (mitogenome) architecture in angiosperms exhibits substantial interspecies variability [12, 13]. In contrast to the animal counterparts, angiosperm mitogenomes are characteristically larger and more complex [14, 15]. The size of the angiosperm mitogenome varies extensively, exemplified by *Silene conica* [16] possessing the largest known mitogenome at 11.3 Mb, and *Viscum scurruloideum* [13] has the smallest mitogenome (66 kb). Typically, the size range of angiosperm mitogenomes falls between 200 kb and 750 kb. This significant structural diversity among different angiosperm mitogenomes is largely attributed to the prevalence of repetitive sequences. These sequences facilitate homologous recombination, which is a crucial mechanism for maintaining genomic integrity, enhancing genomic diversity, propelling genomic evolution, and facilitating adaptation to environmental changes [17]. The frequency, location, and type of these recombination events significantly influence the structure and evolutionary path of the genome [18]. Advancements in high-throughput sequencing technologies have revolutionised our understanding of plant mitochondrial genome structures. The conventional model of a primary circular molecule [19, 20] has evolved to a more dynamic concept, encompassing a mix of circular and non-circular forms [21, 22]. For instance, while species like *Pereskia aculeata* [23], *Viburnum chinshanense* [24] and *Panax ginseng* exhibit a master circular mitogenome, others such as *Amorphophallus albus* [25] present a mitogenome comprising nineteen circular molecules, and *Lactuca sativa* [26] exhibits a simple, branched linear mitogenome. In the context of the Cabombaceae family, the mitogenomic data have been relatively unexplored, and the mitogenome of *Nymphaea colorata* representing the first reported within the Nymphaeales order.

This study is the first to describe the *B. schreberi* mitogenome, adding valuable data for the Cabombaceae family and the Nymphaeales order. It provides important ideas for recognizing and studying *B. schreberi*, helping to develop better breeding and farming methods. Also, it supports the idea that plant mitogenomes can exist in different forms, which helps us understand more about the genetic makeup of flowering plants.

## Materials and methods

### Plant materials and the sequencing of mitogenome

Mature, ripe foliar specimens of *B.schreberi* were collected from ShiZhu, Chongqing, China (30°9'N, 108°26'E) and had been archived in the Southwest University. And the accession number is 20210425CC. The total genomic DNA of *B. schreberi* was extracted utilizing the CTAB method [27]. After the extracted DNA pass inspection, they are randomly fragmented using a ultrasonicator. This is followed by end repair, A-tailing, sequencing adapter ligation, purification, and PCR amplification to complete the entire Illumina library preparation process [28]. And then we sequenced on the NovaSeq-6000 platform, resulting in generation of approximately 12.45 Gb of raw data. We processed the clean data using Trimmomatic [29] which involved discarding sequences where more than 50% of bases had a quality value lower than Q19, and the sequences data contained more than 5% 'N' bases. Meanwhile, the identical plant samples used for Illumina short read sequencing were also sequenced using the Oxford Nanopore platform. For building the Nanopore library, we first check the purity, concentration, and integrity of the extracted DNA. Then, we build the library using the SQK-LSK109 ligation kit. The data quality control was conducted using Nanoplot (version 1.18.2), particularly focusing on the exclusion of data with a quality value below Q7. The Oxford Nanopore sequencing yielded a total of 7.52 Gb encompassing 444,069 reads. Post-quality control, 417,360 reads were retained for further analysis.

### The assembly of organelle genome

**Fig. 1:**
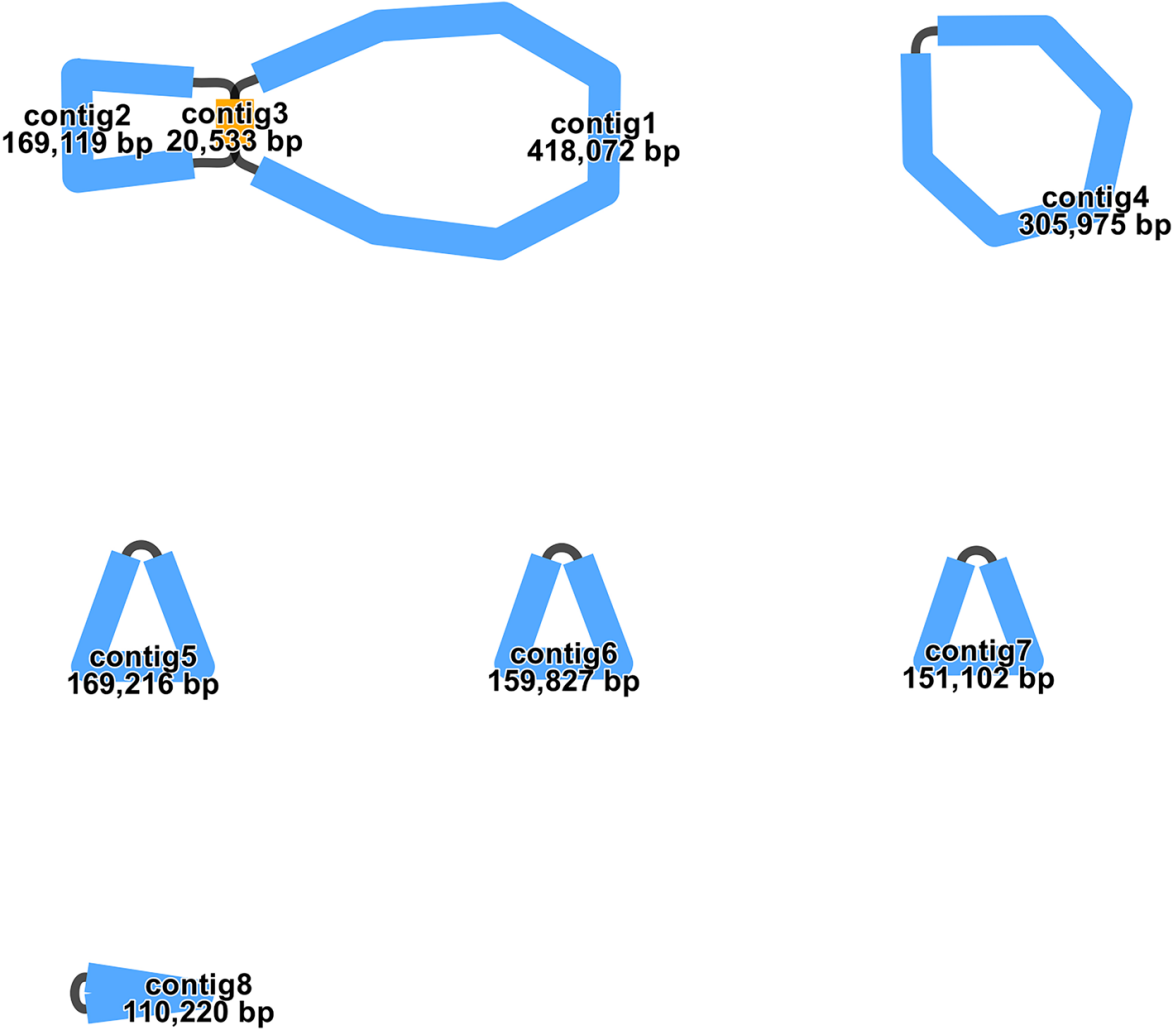
The graphic assembly of *B. schreberi* mitogenome. The graphic mitogenome consists of eight contigs with different lengths

To constructing the *B. schreberi* chloroplast genome, we employed the GetOrganelle tool (version 1.7.4.1). This process involved using specific parameters which were applied to process the Illumina platform short-read sequences [30]. The specific parameters were ‘-R 15 -k 21,45,65,85,105 -F embplant_pt’. This process led to the production of two complete chloroplast genome sequences. Among these, we chose one sequence that showed an alignment of the SSC region consistent with that observed in *Arabidopsis thaliana.* The chloroplast genome of *B. schreberi is* 158,973 bp, and GC content is 39.04%. In assembling the mitogenome, we conducted a de novo assembly of the *B. schreberi* long reads utilizing the Flye tool (version 2.9.1). This involved the application of the parameter setting ‘--min-overlap 2,000’. We identified the mitogenome from the assembling sequences through BLASTn [31]. This process involved creating a sequence database using the makeblastdb and using mitogenome genes from *N. colorata* (NC_037468.1) as the lookup sequences. The approach used was effective in identifying contigs that contained the specified genes. The selection criteria for contigs included the presence of a single homologous sequence region exceeding 2,000 bp or a substantial number of matching hits, with a preference for contigs labeled as loops in the assembly outcomes. This process successfully identified eight mitochondrial contigs (Fig. 1). Subsequent steps involved mapping both short reads and long reads to contigs using BWA and SAMTools [32, 33], retaining all mapped reads. We speculated that certain mitogenome regions may have been inadvertently replaced by their corresponding chloroplast sequences during the refinement phase, due to possible homology between the two. For this purpose, we adopted a hybrid assembling strategy using Unicycler [34], integrating both short reads of Illumina platform and long reads of Nanopore platform. Initially, the short reads of Illumina platform were assembled via SPAdes [35]. Subsequently, the long reads of Nanopore platform were employed to resolve repetitive sequences in the assembly, a process aided by minimap2 [36].The Graphical Fragment Assembly (GFA) files, produced through Unicycler, were visualized using Bandage software [37]. The Unicycler hybrid assembly produced eight contigs, which matched the initial Flye assembly. Notably, the use of Illumina short reads to assemble these contigs eliminated the need for further refinement steps.

### Mitogenome and chloroplast genome annotation

The chloroplast genome of *B. schreberi* underwent annotation employing the CPGAVAS2 platform [38], with the chloroplast of *Cabomba aquatica* (NC_039434.1) serving as the reference genome. To validate the accuracy of these annotations, the CPGView tool [39] was subsequently utilized for verification. To annotate the assembled mitogenome of *B. schreberi*, we utilized the GeSeq tool [40], with reference to two mitogenomes from the GenBank database. The mitogenome of *Liriodendron tulipifera* (NC_021152.1) was selected as the primary reference due to its comprehensive mitochondrial gene annotations, which are among the most detailed for angiosperms. The secondary reference for the annotation was the mitogenome of *N. colorata* (NC_037468.1), distinguished as the first documented mitogenome within the Nymphaeales order. Additionally, we employed the recently developed tool IPMGA (http://www.1kmpg.cn/ipmga/) for annotation and integrated the results from both tools. IPMGA performed well in identifying gene splice sites, especially for *trans*-spliced genes. For the annotation of tRNA genes, we employed tRNAscan-SE [41]. rRNA annotations were determined using BLASTn [31]. To enhance the precision of these annotations, manual adjustments were implemented using the Apollo software [42]. The final phase of the process involved generating the genome map, which we accomplished using the OGDRAW software (version 1.3.1) [43]. This comprehensive approach ensured a robust and accurate annotation of the *B. schreberi* chloroplast genome and mitogenome, contributing valuable genomic data for further research in plant genomics.

### Analysis of codon usage

In our study, the PhyloSuite software (version 1.2.2) [44] was utilized for processing the GenBank format files corresponding to the mitogenome of *B. schreberi*. This processing involved the extraction of PCGs from the mitogenome data. Following this extraction, we undertook a detailed analysis of codon usage patterns within these mitochondrial PCGs. This analysis was conducted using the Mega 7.0 software [45], and it primarily focused on the computation of Relative Synonymous Codon Usage (RSCU) values. This method allowed for a thorough examination of the codon usage biases within the mitochondrial PCGs of *B. schreberi*, contributing to a deeper understanding of its genetic coding efficiency and patterns.

### Repetitive elements

In our study, the detection of long tandem repeats (LTR) within the assembled mitogenome was executed using the Tandem Repeats Finder (TRF) tool, following its default parameter settings. To detect the presence of Simple Sequence Repeats (SSRs) within the *B.schreberi* mitogenome, the web service of MISA was utilized. We configured its parameters to define the minimum number of occurrences for various types of nucleotide repeats: mononucleotide repeats at least 10 times, dinucleotide repeats a minimum of 5 times, trinucleotide and tetranucleotide repeats at least 4 times, and pentanucleotide and hexanucleotide repeats a minimum of 3 times each. Additionally, the task of identifying forward, reverse, complementary and palindromic repeats in the mitogenome was carried out using the REPuter tool [46]. The parameters established for this analysis were a distance of 3, a minimum repeat sequence size of 30 bp, and an e-value threshold set at less than 1e-05. The repetitive sequences identified through these methods were subsequently visualized using the Circos software package [47]. This comprehensive approach to repetitive sequence analysis in the *B. schreberi* mitogenome allows for a detailed characterization of its structural components.

### Verification of the chromosome 1 structure

Based on our assembly results, we numbered them as Chromosome 1 to Chromosome 6 according to the length of each chromosome (Table 1). During the assembly and analysis of the mitogenome of *B. schreberi*, a significant long forward repeat sequence was identified in chromosome 1. This discovery emerged as a notable feature in our study of the genome's repetitive sequences. This sequence is thought to facilitate recombination processes within the chromosome. To investigate this recombination phenomenon, polymerase chain reaction (PCR) experiments were conducted. For these experiments, we developed four unique primers, each specifically designed to target distinct regions (Figure S 1A) to substantiate the recombination events. Primer 1 was designed to validate the path 1 (p1), Path 2 through 4 (p2-p4)each have corresponding primers (primer 2-primer 4) for validation. The design of the primers was carried out using the Primer Designing Tool on the National Center for Biotechnology Information (NCBI) website. This process was carried out using the standard parameter settings provided by the tool. The sequences of these primers, employed in the PCR assays. DNA extractions were conducted, followed by amplification using the Pro-Flex PCR System. Each PCR reaction was prepared with a total volume of 50 µL, including 1 µL of DNA, 2 µL of each primer, 25 µL master mix, and 20 µL of deionized water. The amplification protocol entailed an initial denaturation at 94℃ for 5 minutes, followed by 30 cycles of 94℃ denaturation for 30 seconds, annealing at 58℃ for 30 seconds, and extension at 72℃ for 60 seconds, concluding with a extension at 72℃ for 5 minutes. The PCR products were then visualized using 1% agarose gel electrophoresis, facilitating the investigation of recombination dynamics within chromosome 1 of the *B. schreberi* mitogenome.

**Table 1:** The information mitogenome of *B. schreberi*

Mitogenome	Length (bp)	GC content (%)	Accession Number
Chromosome 1	628,257	48.78	MZ919331
Chromosome 2	305,975	48.84	MZ919332
Chromosome 3	169,216	48.80	MZ919333
Chromosome 4	159,827	49.54	MZ919334
Chromosome 5	151,102	48.36	MZ919335
Chromosome 6	110,220	49.17	MZ919336

### The Identification of homologous sequences between organelle genomes (MTPTs)

The process of identifying homologous sequences within the two assembled organellar genomes was carried out using the BLASTn program [31]. This task was performed adhering to specific parameters: ‘-1*e*-5, -word size 10, -gapopen 5, -outfmt 6’. The analytical process was specifically designed to determine sequence similarities between the mitogenome and the chloroplast genomes. The visualization of the resulting data was facilitated using the “Advanced Circos” module within the TBTools software suite [48]. Additionally, the GeSeq tool was employed for the annotation of the identified mitochondrial-plastid transition sequences (MTPTs). Considering the placement of the MTPTs within the inverted repeat regions of the chloroplast genome, it was decided to consider each MTPT only once in our analysis. This approach ensures a more accurate representation and analysis of the homologous sequences present between the mitochondrial and plastid genomes, thereby providing a detailed insight into the intergenomic interactions within the assembled organelle genomes.

### Prediction of RNA editing sites

In our research, we utilized the Deepred-mt tool [49], a system grounded in a convolutional neural network model, for predicting cytidine to uridine of RNA editing events within *B. schreberi* mitogenome. This predictive procedure entailed extracting all mitochondrial PCGs from the assembled *B. schreberi* mitogenome. These extracted genes were then inputted into the Deepred-mt system for analysis. The predictions produced by this tool were deemed reliable and selected for further examination only if they showed probability values exceeding 0.9. This threshold was established to ensure a high degree of confidence in the predicted RNA editing sites, thereby enhancing the accuracy and reliability of our genomic analysis of RNA editing within the mitochondrial genome.

### Colinear analysis

For our study, we obtained the mitogenome sequences of several species from the NCBI: *Amborella trichopoda* (KF754799.1 to KF754803.1), *N. colorata* (NC_037468.1), *Schisandra sphenanthera* (NC_042758.1), *Magnolia biondii* (NC_049134.1), and *L. tulipifera* (NC_021152.1). Additionally, we downloaded second and third generation genome sequencing data of *Euryale ferox* from the Sequence Read Archive (SRA) database (SRR5145564, SRR11262220) and proceeded to assemble the *E. ferox* mitogenome. Including the mitogenome of *B. schreberi*, a total of seven mitogenomes were utilized for colinear analysis. The process of identifying colinear blocks within these mitogenomes was grounded in sequence similarity. This was determined by using the BLASTn, employing specific parameters: -1e-5, -word size 9, -gapopen 5, -gapextend 2, -reward 3, -penalty -2. For downstream analysis, we only considered colinear blocks that were greater than 500 bp in length. The multiple synteny plots, illustrating genomic collinearity, were generated using TBtools, specifically calling upon the MCscan source program for this purpose. Moreover, self-dotplots for *B. schreberi* and comparative dotplots between *B. schreberi* and the other six mitogenomes were created employing the MAFFT alignment tool. The dotplot serve to graphically represent genomic alignments and similarities, thereby providing a clear comparative genomic perspective of *B. schreberi* in relation to the selected angiosperm species.

### Phylogenetic analysis

Utilizing the conserved PCGs from the mitogenome, we conducted a comprehensive genomic analysis. For this purpose, the mitogenomes of 36 species closely related to *B. schreberi*, along with one outgroup species, *A. trichopoda*, were downloaded from the NCBI Nucleotide database. This selection was based on the phylogenetic proximity to *B. schreberi*. The extraction and identification of 24 orthologous PCGs across these species were accomplished using PhyloSuite software (version 1.2.2) [44]. Following this, we used the MAFFT software (version 7.471) [50] to align these PCGs sequences. The alignment of these sequences enabled their concatenation, which served as the foundation for constructing a phylogenetic tree. For this purpose, the Maximum Likelihood (ML) phylogenetic analysis was carried out using RAxML software (version 8.2.4) [51]. The computational analysis employed the following settings: execution through ‘raxmlHPC-PTHREADS-SSE3’ with the options ‘-f a -N 1000 -m GTRGAMMA’, alongside the seeds '-x 551314260' and '-p 551314260' for random number generation. Additionally, to ensure the robustness of the phylogenetic tree, a bootstrap analysis with 1,000 replicates was performed. The resultant phylogenetic tree was then refined and visualized by ITOL [52], and nodes (100/1) that are fully supported are not marked.

## Results

### Structural characteristics of organelle genome

In our study, we had successfully assembled six complete circular DNA molecules constituting the mitogenome of *B. schreberi*, as illustrated in Fig. 1. Utilizing second-generation sequencing data, these six circular molecules were observed to form an integrated closed-loop structure. This observation leads to the hypothesis that a substantial number of repetitive sequences may be facilitating recombination events within the *B. schreberi* mitogenome. Although third-generation sequencing data corroborate the existence of these six independent circular structures, the actual configuration of the *B. schreberi* mitogenome may be considerably more intricate. Our assembly, representing six circular molecules, potentially exemplifies just one of several possible structural states. It is important to note that there is no physical mapping evidence to definitively support the existence of these six independent molecules. Therefore, the use of the term “chromosome” in this context is a convention adopted for the ease of describing our assembly results and should not be directly equated with chromosomes of the nuclear genome. The size of these six mitochondrial chromosomes ranges from 628,257 bp to 110,220 bp, with a cumulative length of approximately 1.49 Mb. The GC content of these chromosomes varies between 48.36% and 49.54%. The specific details of the length and GC content of each chromosome in the *B. schreberi* mitogenome are provided in Table 1. To confirm the accuracy of our mitogenome assembly, we mapped Illumina short-reads onto the assembled structure. This process achieved an average depth of coverage of approximately 80-fold, as illustrated in Figure S 1. This comprehensive coverage across all six chromosomes strongly suggests the accuracy of our assembly results. During the assembly process, we discerned a significant long forward repeat sequence in chromosome 1. It is theorized that this sequence may function in facilitating recombination within the chromosome. This particular aspect will be further explored and discussed in the results section of our study.

### Mitogenome annotation

**Fig. 2:**
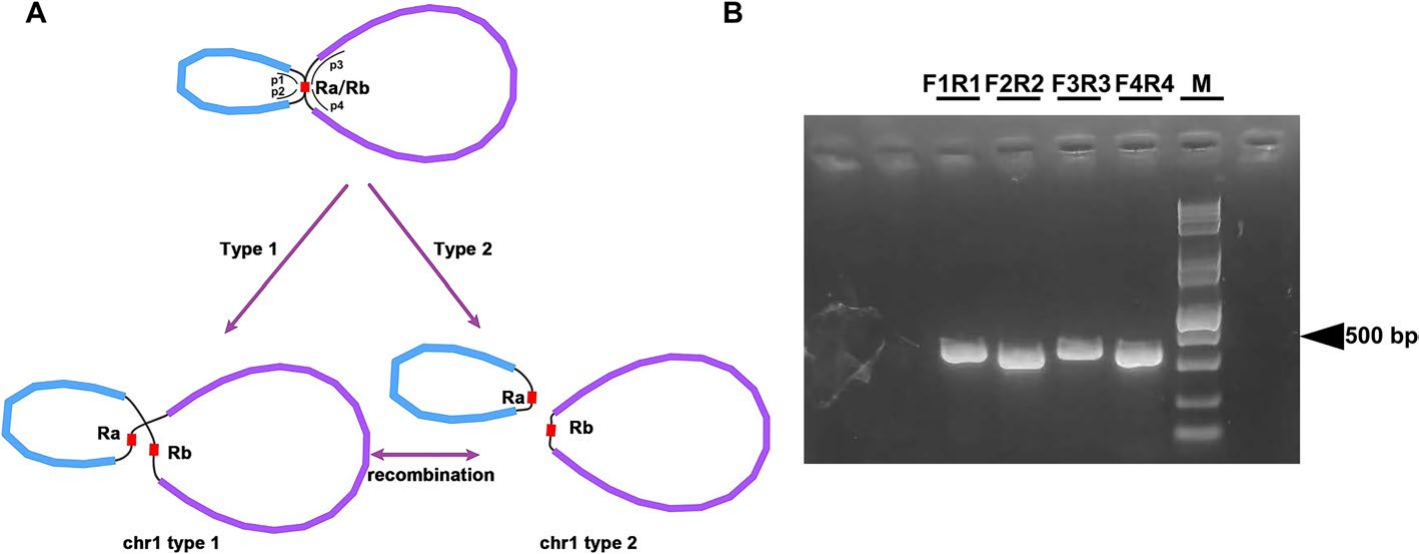
The verification of recombination in chromosome 1. **A** showed the two types of recombination in chromosome 1 and the four paths. **B** showed the electropherogram for these four paths

The mitogenome of *B. schreberi* has been comprehensively annotated, revealing the presence of 40 PCGs (Fig. 2). These include 24 core genes, encompassing fundamental mitochondrial functions, and 16 variable genes, which exhibit diversity in their presence across species. The details of these genes are enumerated in Table S 1. The core genes of the studied genome consist of a suite of ATP synthase genes (*atp1, atp4, atp6, atp8, atp9*), a group of nine NADH dehydrogenase genes (*nad1, nad2, nad3, nad4, nad4L, nad5, nad6, nad7*, and *nad9*), and four genes integral to Cytochrome C biogenesis (*ccmB, ccmC, ccmFC, ccmFN*). Additionally, the genome contains three Cytochrome C oxidase genes (*cox1, cox2, cox3*), a gene for a membrane transport protein (*mttB*), a maturation enzyme gene (*matR*), and a gene encoding Panthenol-cytochrome C reductase (*cob*). The variable gene set includes four ribosomal large subunit genes (*rpl2, rpl5, rpl10, rpl16*), ten ribosomal small subunit genes (*rps1, rps2, rps3, rps4, rps4_copy2, rps10, rps11, rps12, rps13, rps14, rps19*), and two Succinate dehydrogenase genes (*sdh3, sdh4*). Through tRNAscan-SE analysis, we annotated 28 tRNA genes, including eight unique ones. Importantly, ten tRNA genes (*trnC-GCA, trnM-CAU, trnI-CAU, trnQ-UUG, trnN-GUU, trnT-GGU, trnW-CCA, trnA-UGC, trnI-GAU,* and *trnV-GAC*) are hypothesized to originate from the plastid genome. Additionally, three unique ribosomal RNA (rRNA) genes were annotated: *rrn5, rrn18*, and *rrn26*. A peculiar observation was made regarding two genes (*sdh3, sdh4*) that lacked annotated stop codons, suggesting the possibility of them being gene fragments. Intronic analysis within mitochondrial genes revealed introns in nine genes, with varying numbers and distributions. Interestingly, in *B. schreberi*, the exons of three genes that undergo trans-splicing (*nad1, nad2,* and *nad5*) are distributed across different chromosomes. For example, the exons of *nad1* are spread between chromosomes 1, 2, and 3, while *nad2* and *nad5* exons are distributed between chromosomes 1 and 4. The specific lengths and genomic positions of each PCG (exon) are detailed in Table S 1. The accession numbers for each chromosome are provided in Table 1. This intricate arrangement of exons and introns in the *B. schreberi* mitogenome underscores the complexity and uniqueness of its genomic architecture.

### Codon usage of PCGs

**Fig. 3:**
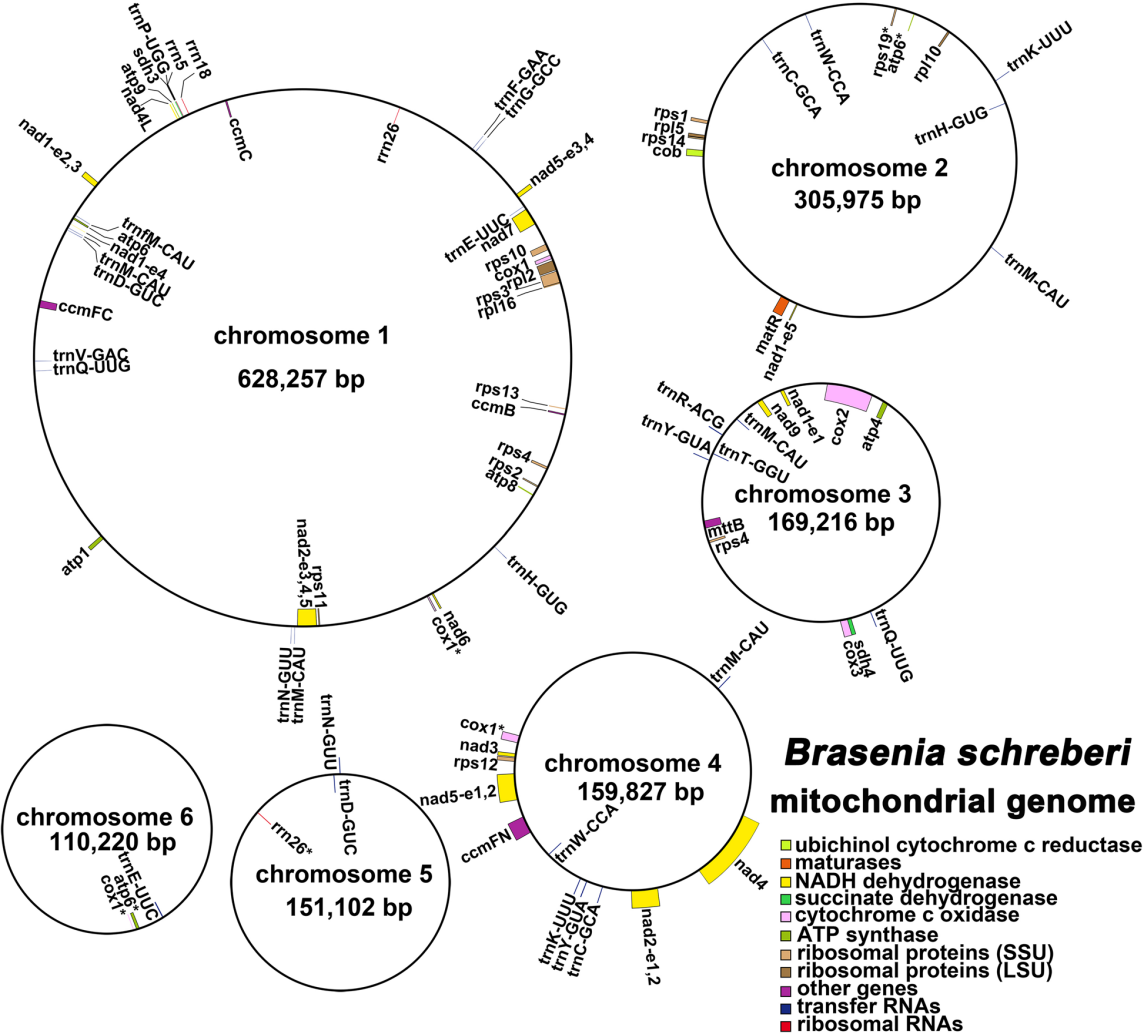
Mitochondrial genome map of *B. schreberi*. The mitogenome consists of six circular chromosomes with different lengths and gene contents. Genes transcript clockwise or counter-clockwise strands are drawn on the upper or lower of the circles, respectively. Genes belonging to different functional groups are color-coded

In our study, we conducted an analysis of codon usage for the 40 PCGs of the *B. schreberi* mitogenome. The distribution of codon usage across each amino acid is systematically presented in Table S 2. Our analysis revealed a distinct codon usage bias within the PCGs of the *B. schreberi* mitogenome. For example, in the context of the mitogenome PCGs, alanine showed one pronounced preference for GCU, which achieved the highest RSCU value at 1.54. This was closely followed by the preference of tyrosine for the codon UAU, which had the RSCU of 1.52. In addition, three codons showed an RSCU value of 1.5: the termination codon UAA, the codon CAU of histidine and the codon CAA of glutamate. As illustrated in Fig. 3, a pronounced codon usage pattern was observed for each amino acid encoded by the mitogenome PCGs. The average RSCU value for the most frequently used codons was calculated at 1.36, with the exception of two amino acids, methionine (Met) and tryptophan (Trp), which do not have synonymous codons. This analysis provides insight into the codon usage patterns and preferences within the mitochondrial genome of *B. schreberi*, enhancing our understanding of its genetic encoding and expression mechanisms.

### Repeat elements

In our study, we conducted a detailed analysis of three distinct categories of repetitive elements within the mitogenome of *B. schreberi*, revealing a significant prevalence of these sequences. Our study identified 553 SSRs within the *B. schreberi* mitogenome (Table S 3). The majority were tetranucleotide SSRs, totaling 182. Additionally, trinucleotide SSRs were also notably present, amounting to 144. Mononucleotide SSRs were comparatively less frequent, with a count of 46. The analysis also uncovered 82 dinucleotide SSRs, 87 pentanucleotide SSRs, and 12 hexanucleotide SSRs. Then we detected 608 LTRs (Table S 4), characterized by their extended lengths. Of these, 39 tandem repeat units exceeded 100 bp, with the longest extending to 296 bp. These LTRs predominantly reside in chromosomes 1 to 4, with a smaller presence in chromosomes 5 and 6. Meanwhile, we enhanced the size criterion for minimum repeat length to 100 bp, we identified 1,822 dispersed repeats in the *B. schreberi* mitogenome (Table S 5). This group comprised 1,015 forward repeats and 807 palindromic repeats. A significant number of these repeats are distributed across various chromosomes, suggesting they may contribute to the segmentation of the *B. schreberi* mitogenome into six distinct molecular entities.

**Fig. 4:**
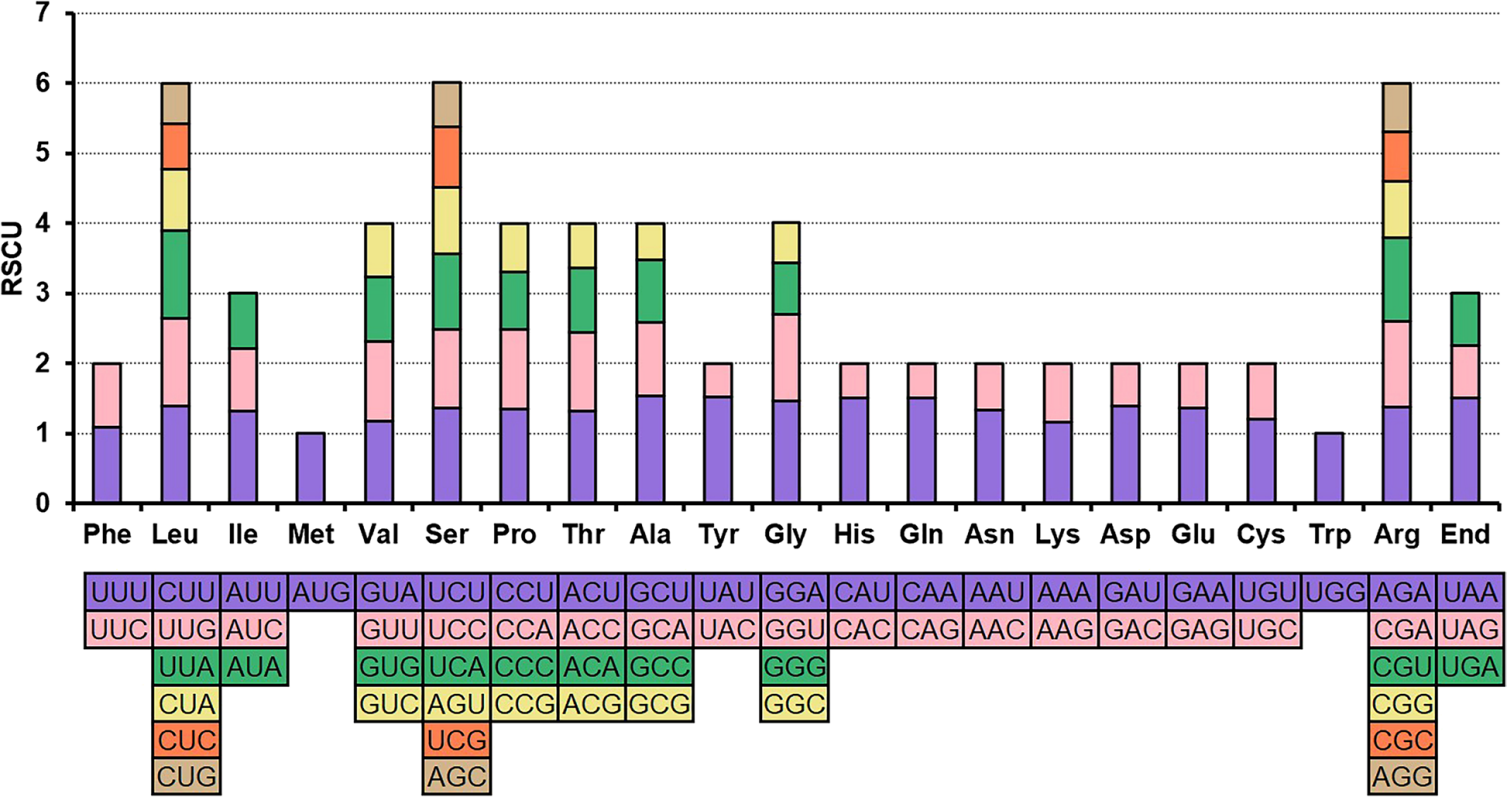
Codon usage bias of mitochondrial PCGs of *Brasenia schreberi*. Codon families are shown on the x-axis. RSCU values are the number of times a particular codon is observed relative to the number of times that codon would be expected for uniform synonymous codon usage

**Fig. 5:**
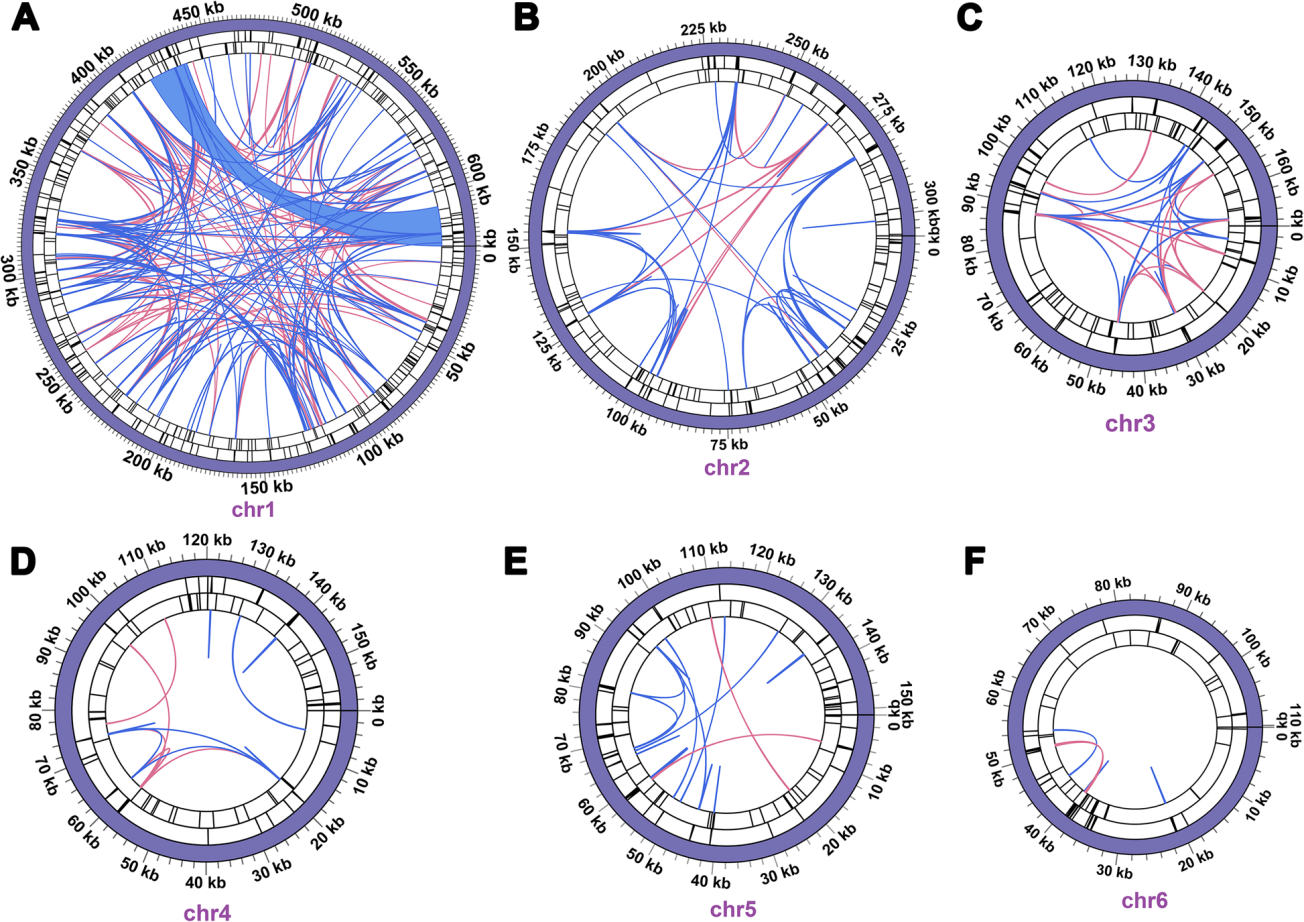
Distribution of different repeats on each chromosome of the *B. schreberi* mitochondrial genome. A-F were chr1 to chr6, respectively. The dispersed repeats, SSRs, LTRs and the relative positions of mitogenomes are shown from inside to outside. Among them, the innermost arc connects the two dispersed repeated units, with red representing palindromic repeats, blue representing forward repeats

The distribution of these repetitive elements within the chromosomes is depicted in Fig. 4. Notably, a long forward repeat, measuring 20,545 bp, was identified on chromosome 1 (Fig. 4A), likely playing a pivotal role in its cleavage. Figure 5 graphically represents the distribution of dispersed repeats across various chromosomes. Of the 1,822 dispersed repeats, 1,291 have repeat units distributed between distinct chromosomes. These extensive dispersed repeats, exceeding 100 bp in length, imply a probable interconnection among the various chromosomes, elucidating why the Illumina-based assembly depicted a complex, unified structure instead of six independent molecules. Consequently, the *B. schreberi* mitogenome is inferred to be dynamic in nature, possessing a diverse subgenomic architecture.

### Verification of recombination of chromosome 1

**Fig. 6:**
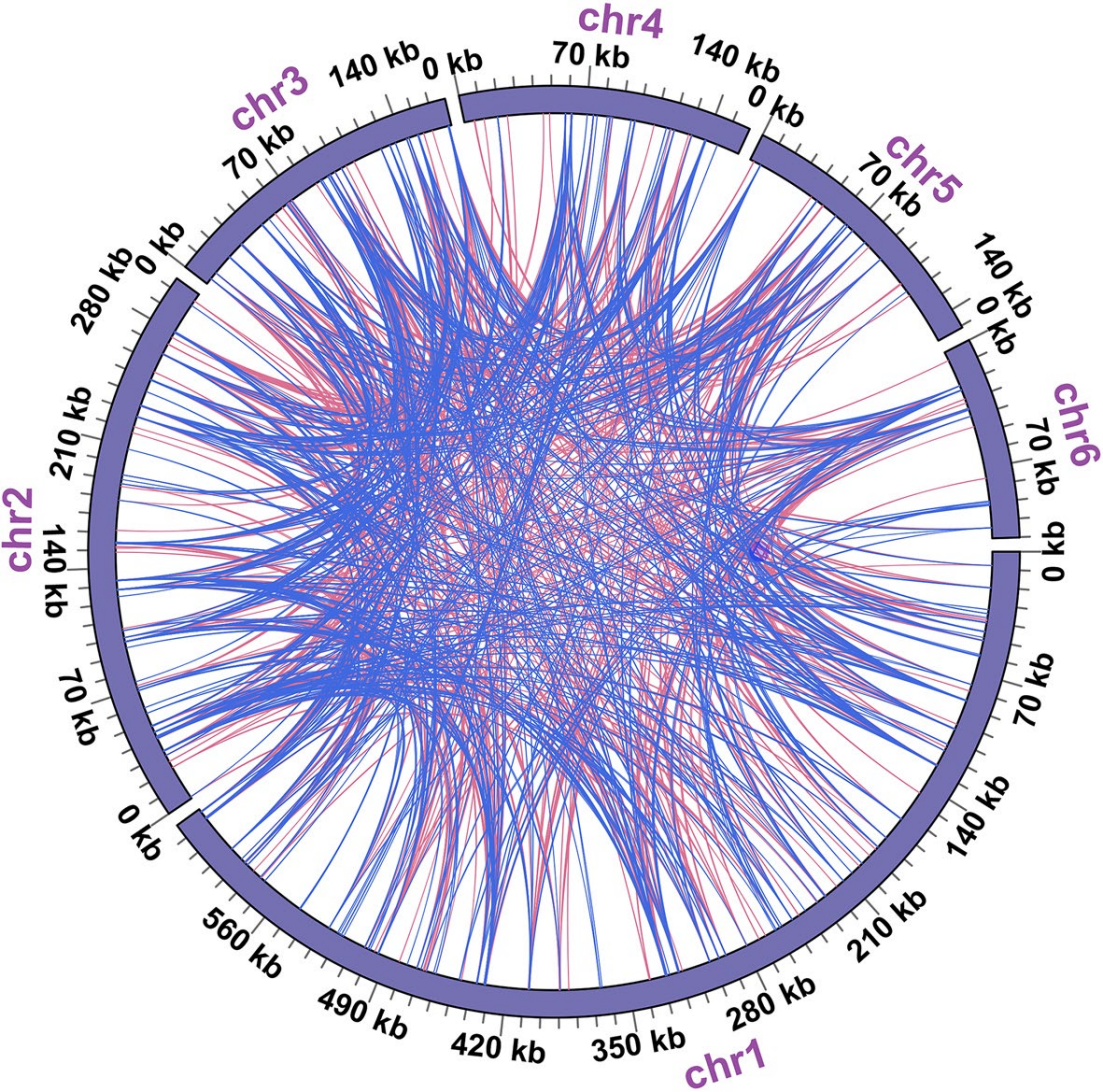
Schematic diagram of dispersed repeats between different chromosomes in the *B. schreberi* mitochondrial genome. The arc connects the two dispersed repeated units, with red representing palindromic repeats, blue representing forward repeats

During the assembly of the *B. schreberi* mitogenome, we employed Bandage for the graphical representation of the assembled mitogenome. Notably, chromosome 1 was represented by three distinct nodes, as illustrated in Fig. 6A. Within this structure, contig 3 was identified as a long forward repeat sequence, potentially playing a crucial role in mediating recombination events within chromosome 1. This observation led us to hypothesize two potential genomic configurations for chromosome 1: (1) a complete master circular configuration (type 1), and (2) a configuration where “chromosome 1” is divided into two smaller loops facilitated by the aforementioned repeat (type 2), as depicted in Fig. 6A. The repeat region's considerable length posed a challenge, as long reads sufficient to span this region were not available even in the ONT sequencing data. As shown in Fig. 6A, the potential for the formation of type 1 configuration arises through the mediation of the long forward repeat sequence from p1 to p4 and from p2 to p3. Conversely, the formation of type 2 configuration could be facilitated by the same repeat sequence from p1 to p3 and from p2 to p4. To confirm the accuracy of two hypothesized configurations of *B. schreberi* PCR experiments were performed. Four primer pairs were specifically designed and utilized to validate the paths p1, p2, p3, and p4, respectively (Fig. 6A). The PCR amplification yielded bands corresponding to the expected sizes (Fig. 6B), corroborating the plausibility of both genomic configurations. The unprocessed electropherogram of these results is provided in Figure S 2. For the purposes of our analysis, we have decided to utilize this structure of the complete master circular chromosome 1 in subsequent investigations.

### Analysis of mitochondrial plastid DNA sequences (MTPTs)

**Fig. 7:**
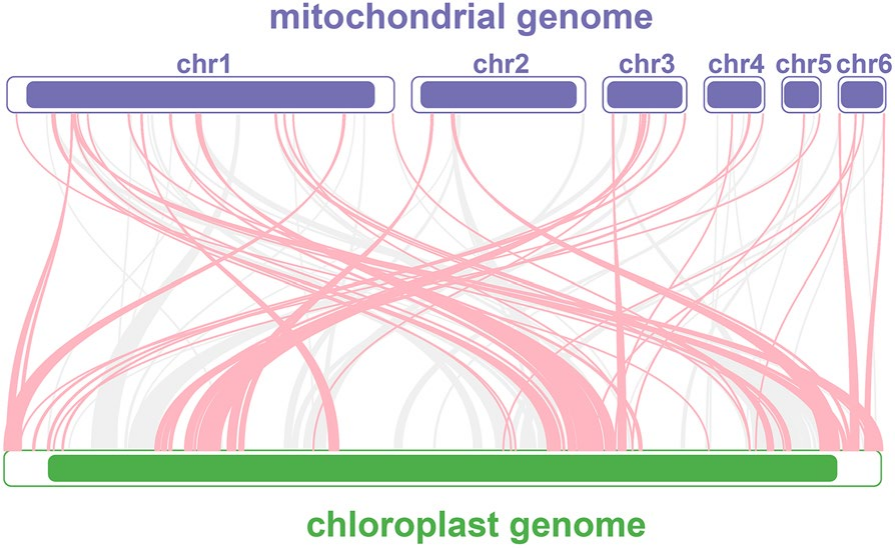
Schematic diagram of homologous sequence between mitochondrial genome and chloroplast genome of *B. schreberi*. The red curves indicate the opposite direction (inverted) and the gray curves indicate the same direction

**Fig. 8.**
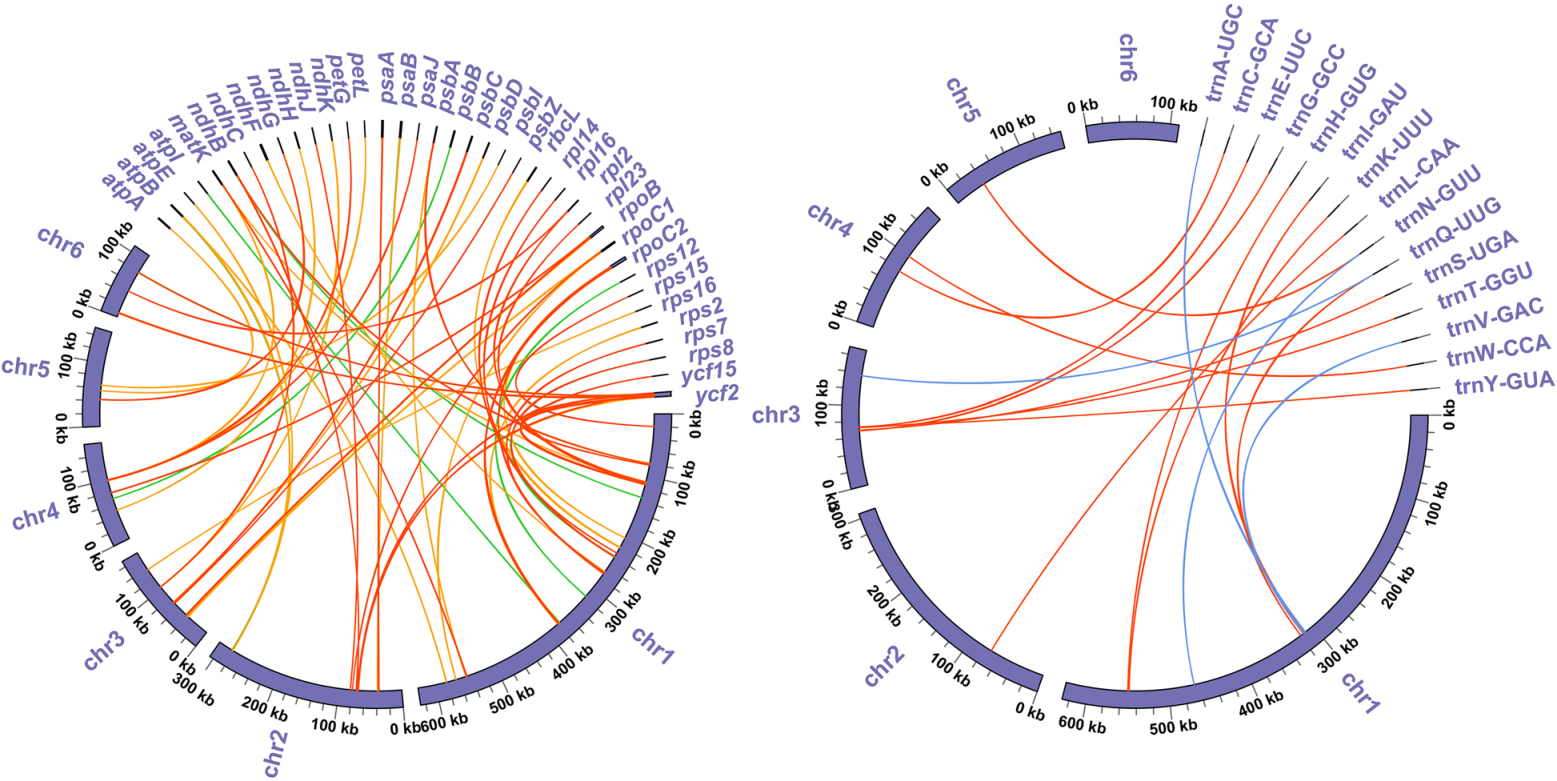
Visualization of BLAST results betwwen chloroplast gene and mitochondrial genome in *B. schreberi*. Red arcs indicate sequence similarity of more than 90%, orange arcs indicate sequence similarity of more than 80%, green arcs indicate sequence similarity of more than 70%, and blue arcs indicate sequence had 100% similarity

In *B. schreberi*, there is a notable exchange of sequence between the chloroplast genome and the mitogenome. As depicted in Fig. 7, all six mitochondrial chromosomes of *B. schreberi* harbor sequences homologous to the chloroplast genome. The extent of this homology is substantial, with approximately 57 kb of chloroplast DNA exhibiting similarity to the mitogenome. This represents nearly 36.1% of the total chloroplast genome and approximately 3.8% of the mitogenome, suggesting that the chloroplast genome serves as a significant source of extraneous DNA for the mitogenome of *B. schreberi*. In our detailed analysis, we focused on homologous sequences exceeding 500 bp in length. As shown in Table S 6, the most extensive of these homologous sequences spans 3,906 bp, located on chromosome 1 (MTPT1). Additionally, two sequences exceeding 2,000 bp were identified: a 2,237 bp sequence on chromosome 2 (MTPT2) and a 2,044 bp sequence on chromosome 1 (MTPT3). Upon annotation of these homologous sequences, it was found that many still contain fragments of chloroplast genome genes, such as *ycf2, rbcL, psaB, psbA, rpoC2*, and others. However, these gene sequences are incomplete, likely representing remnants of once-transferred segments. Previous research indicates that these chloroplast-derived genes do not retain functional roles and are progressively evolving into pseudogenes. To further determine which chloroplast genes are present in the *B. schreberi* mitogenome, we employed the BLASTn program to specifically search for homologous sequences of chloroplast PCGs and tRNA genes (Fig. 8). As illustrated in Table S 6, a total of 39 chloroplast PCGs and ten tRNA genes were identified in the *B. schreberi* mitogenome. In contrast to the PCGs, which are entirely fragmented, the ten tRNA genes were found to have sequences completely identical to their chloroplast counterparts. These tRNA genes are *trnC-GCA, trnM-CAU, trnI-CAU, trnQ-UUG, trnN-GUU, trnT-GGU, trnW-CCA, trnA-UGC, trnI-GAU,* and *trnV-GAC*. Based on their sequence integrity, we propose that these four mitochondrial tRNA genes likely originated from chloroplast gene transfer.

### The Prediction of RNA editing events

**Fig. 9:**
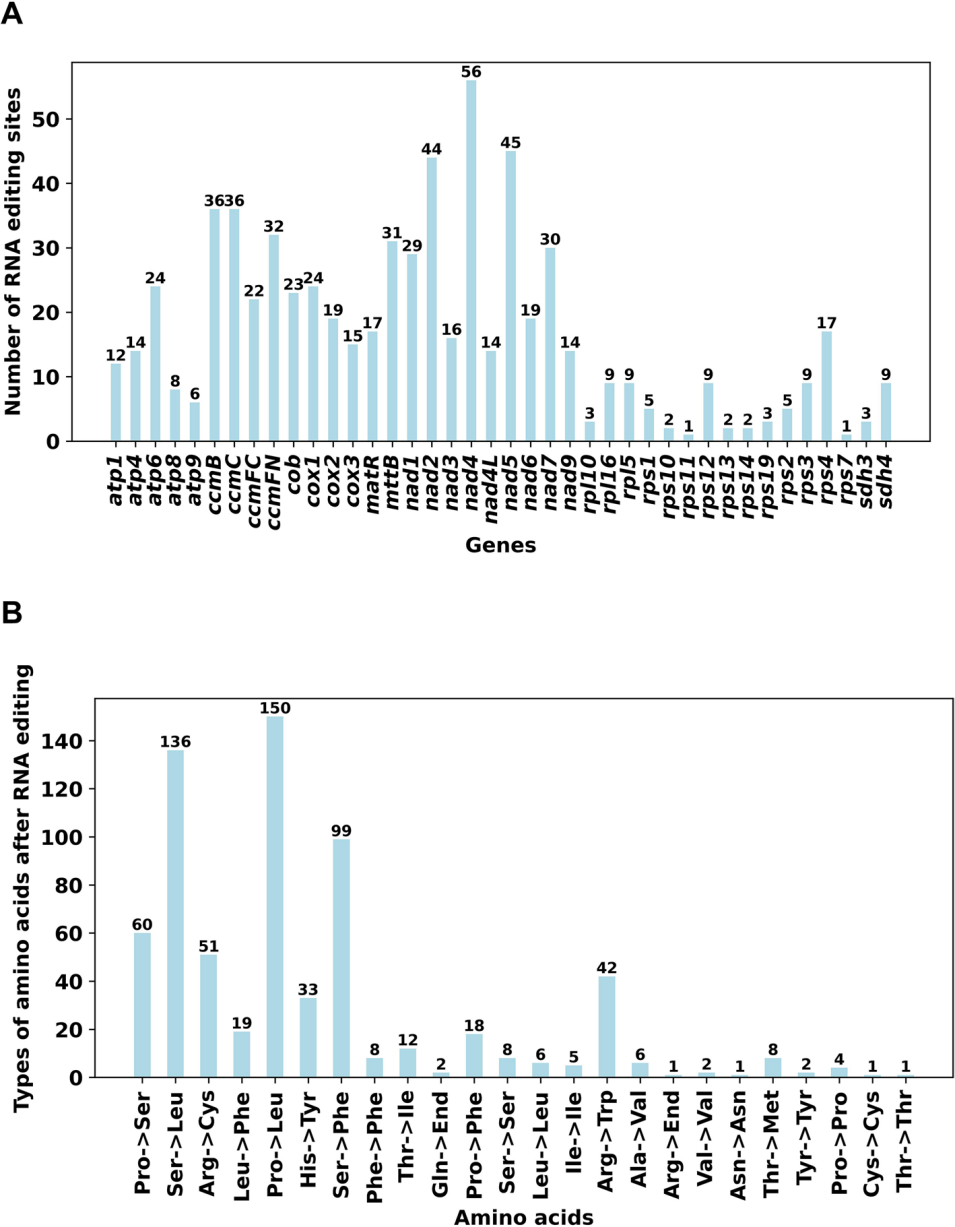
Characteristics of the RNA editing sites identified in mitochondrial PCGs of *B. schreberi*. **A**. The ordinate shows the number of RNA editing sites identified in PCGs, the abscissa shows the name of PCGs identified in the mitogenome of *B. schreberi*. Panel **B** shows the potential effect of all RNA editing events on amino acids. Most of these RNA editing events lead to changes in amino acids

Through the application of the Deepred-mt tool, our study identified 675 C to U RNA editing sites of notable confidence within 40 genes of the mitochondrial protein-coding genome, as elaborated in Table S 7. The distribution of predicted RNA editing sites across each gene is visually represented in Fig. 9A. Among these genes, *nad4* is distinguished by the highest count of RNA editing sites, totaling 56, closely followed by *nad5* with 45 sites. Furthermore, a number of genes such as *ccmB, mttB, ccmC, nad2, nad7* and *ccmFN*, each display over 30 RNA editing sites. Conversely, genes like *rps11* and *rps7* show limited RNA editing activity, with only two C to U editing sites identified in each. The analysis by Deepred-mt highlighted RNA editing events in four genes that lead to the formation of premature stop codons. These include the *atp6* gene, where a CAA codon is edited into UAA, and the *ccmFC* gene, where a CGA codon is changed to UGA. In the *rps14* gene, a CAG codon is edited to UAG. Additionally, RNA editing is crucial in creating start codons for several genes, such as *nad1*, *nad4L*, by changing ACG codons to AUG.

Figure 9B illustrates how these RNA editing events affect the subsequent amino acid translations. Among all the identified RNA editing sites identified, 638 result in non-synonymous changes, thereby altering the amino acids they encode. This accounts for a substantial 94.52% of all editing events. The most frequent amino acid alteration involves the conversion of proline (Pro) to leucine (Leu), occurring in 150 instances. Additionally, serine (Ser) is commonly replaced by leucine (Leu) in 136 cases. These two types of substitutions together comprise 42.37% of all observed amino acid changes. Conversely, synonymous substitutions are relatively infrequent, with only 37 instances identified, underscoring the significant role of RNA editing in modulating protein translation in the *B. schreberi* mitogenome.

### Colinear analysis

**Fig. 10:**
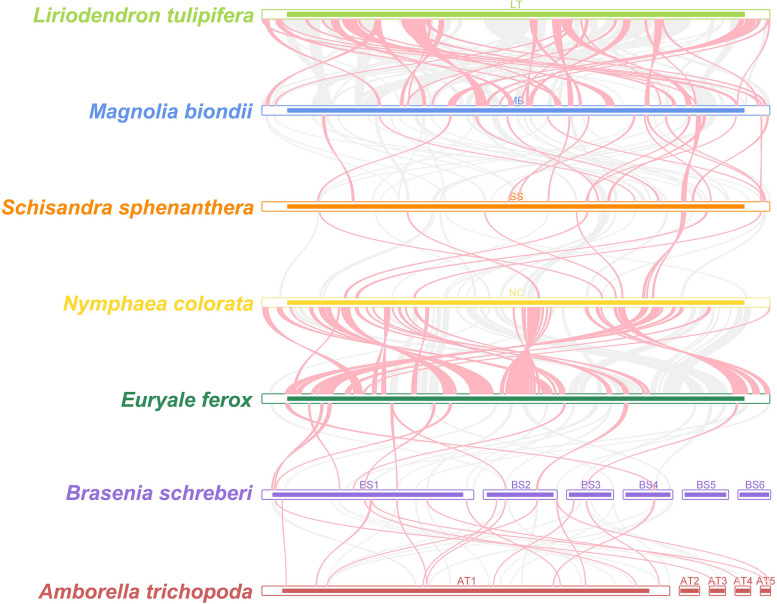
Collinearity analysis between seven mitochondrial genomes. Only collinear blocks over 1,000 bp in length are retained. The red curves indicate the opposite direction (inverted) and the gray curves indicate the same direction

In our study, we performed a comparative analysis to evaluate the structural conservation between the mitogenome of *B. schreberi* and that of six phylogenetically related species. This analysis encompassed the visualization of collinear blocks and the construction of dot plots. As depicted in Fig. 10, the analysis revealed a lack of substantial conserved collinear blocks between *B. schreberi*, *A. trichopoda*, and *E. ferox*. This absence is possibly attributable to significant recombination events that have occurred in the *B. schreberi* mitogenome. Furthermore, the size discrepancy among the mitogenomes of these species is notable, with *A. trichopoda* and *B. schreberi* possessing mitogenomes of over 3 Mb and 1.49 Mb, respectively, in contrast to the considerably smaller mitogenome of *E. ferox*, approximately 370 kb. This size variation has resulted in the integration of numerous species-specific sequences within the mitogenomes of *B. schreberi* and *A. trichopoda*, which lack homology with sequences found in other species. Notably, no significant homologous regions were identified between chromosomes 5 and 6 of the *B. schreberi* mitogenome and the genomes of other species. In the case of Magnoliaceae family members *M. biondii* and *L. tulipifera*, as well as Nymphaeaceae family members *E. ferox* and *N. colorata*, numerous homologous collinear blocks were detected, suggesting that genetic proximity correlates with the retention of more collinear blocks.

The dot plot analysis (Figure S 3) further highlights the presence of multiple divergent regions between the mitogenomes of *B. schreberi* and its closely related species. Conserved collinear regions are sporadically distributed, primarily aligning with conserved mitochondrial gene sequences. This finding indicates that a significant portion of the non-coding regions in the *B. schreberi* mitogenome are distinct and unique. Additionally, the stark variation in mitogenome sizes among these species is noteworthy. Previous research has suggested that the mitogenome of *A. trichopoda* underwent expansion due to the mitochondrial engulfment and retention of genetic material from other plant mitogenomes, resulting in its unusually large size. The non-coding regions of the *B. schreberi* mitogenome are characterized by an abundance of repetitive sequences and exogenous sequences, among which the chloroplast genome of *B. schreberi* might be a predominant source of extraneous genetic material.

### Phylogenetic analysis

**Fig. 11:**
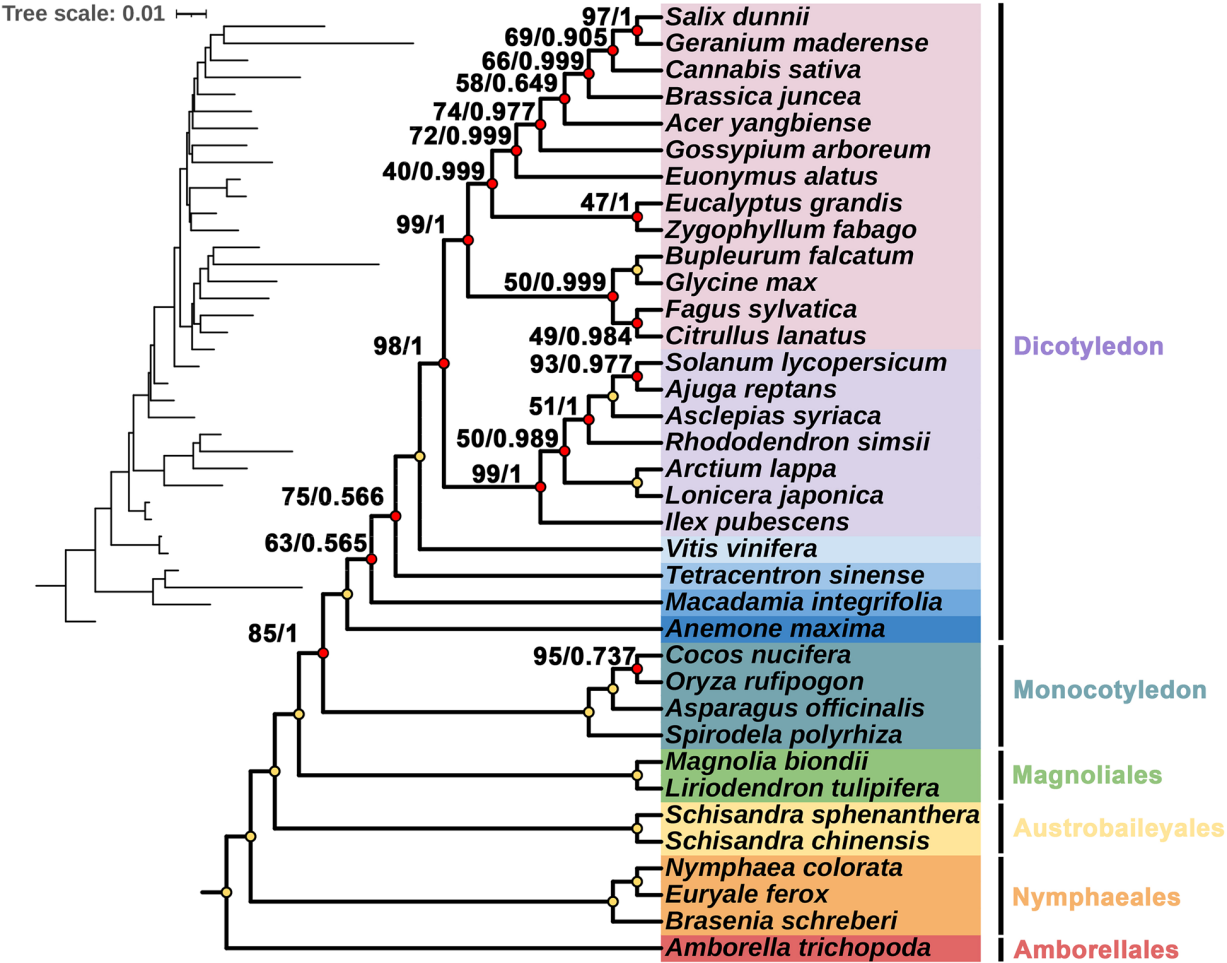
Phylogenetic trees constructed based on mitochondrial DNA. Nodes (100/1) that are fully supported are not marked. The length of the branch represents the frequency of base substitutions at each locus in the genome

Based on the conserved mitochondrial PCGs sequences, we constructed a phylogenetic tree of 36 angiosperm species (Fig. 11). The list of specific plant species can be found in Table S 8. The results showed that *B. schreberi* is more closely related with *Euryale ferox.* The mitochondrial DNA-based phylogenetic tree showed relatively low support, especially for the species at the crown of the tree. However, our tree obtained ultra-high support in several basal branches. This finding was in alignment with the recent classification system of the Angiosperm Phylogeny Group (APG). Nevertheless, the plants at the crown of the tree did not receive high support, and their topology was inconsistent with the APG classification. This observation could be associated with the phenomenon of horizontal gene transfer in plant mitochondrial genes. Previous studies have shown that some mitochondrial genes in plants may have been acquired from other plants, resulting in inconsistent topology with the true phylogeny [53, 54]. Thus, the evolutionary lineage depicted by the phylogenetic tree based on mitochondrial DNA might not precisely mirror the actual evolutionary ties. Given its semi-autonomous genetic framework, mitochondria may display divergent evolutionary trajectories compared to the nuclear or chloroplast genomes [55–57].

## Discussion

### Highly variable mitogenome

Contrasting with the highly conserved chloroplast genome, plant mitochondrial genomes exhibit extreme variability in their genomic structures. Even among closely related species, there are numerous inversions, rearrangements, and incorporations of foreign sequences, leading to a lack of good collinearity in their mitogenomes. Moreover, there is a significant variation in the size of plant mitogenomes. The smallest reported plant mitogenome, from the Viscum plant *Viscum scurruloideum*, is just 66 kb [16], whereas some gymnosperms have mitochondrial genomes exceeding 4 Mb [58, 59]. In angiosperms, for instance, the mitogenome of *A. trichopoda* exceeds 3 Mb, while some Brassicaceae plants like *Brassica oleracea* have genomes as small as 219 kb [60]. This is primarily due to the incorporation of a huge number of foreign sequence in the mitogenome of *A. trichopoda*. The *B. schreberi* mitogenome sequence is over 1.49 Mb, exceeding most published angiosperms. In the Nymphaeales order, this size is 2.4 times that of the previously published *N. colorata* (about 617 kb) and four times that of the *E. ferox* (about 370 kb). This is mainly attributed to redundancy from a huge number of repetitive sequences and the rich absorption of chloroplast DNA from the plant itself. Furthermore, there is variability in the number of genes in plant mitogenomes. Most plant mitogenomes contain 24 conserved PCGs, but ‘variable genes’ such as those for large and small ribosomal subunits, and succinate dehydrogenase, show varying degrees of loss across different species, and it is rare for a plant to have all these variable genes. Within the mitogenome of *B. schreberi*, there are four large ribosomal subunit genes (*rpl2, rpl5, rpl10* and *rpl16*), nine small ribosomal subunit genes (*rps1, rps2, rps3, rps4, rps10, rps11, rps12, rps13, rps14*), and the two succinate dehydrogenase genes (*sdh3* and *sdh4*). Compared to the *N. colorata, B. schreberi* lacks the *rps19* gene, which was detected only as a fragment and possibly a pseudogene. Both succinate dehydrogenase genes are completely lost in some Poaceae plants [61]. In some Lamiaceae plants, there is a greater loss of large and small ribosomal subunit genes, as seen in *Ajuga reptans* [62], where genes *rpl5, rpl10, rpl16, rps7, rps10*, and *rps14* are completely lost, and *rps4* exists only as a fragment. These variable genes vary significantly in number, but the count of core genes is extremely conserved. Current research has only found the loss of some subunits related to the NADH dehydrogenase gene (*nad*-) in the *Viscum* genus [16, 63], but no such loss of these core genes in other parasitic plants like Balanophoraceae [64–66], *Cuscuta* [66], or saprophytic plants like *Gastrodia elata* [67], *Monotropa hypopitys* [68], etc. Homologous recombination, driven by repetitive sequences, is a nearly universal feature in plant mitogenomes. These repeats not only act as prime facilitators for genomic recombination but also substantially contribute to the enlargement of the mitogenome size. Previous studies on the *Lactuca genus* [26] discovered multiple different structures in its mitogenome, including linear, circular, multibranched, and multicircular forms, where repetitive sequences play a crucial role. The *B. schreberi* mitogenome assembly demonstrates a complex map. Although a main existing conformation supported by long-read data was obtained with the help of ONT data (6 circular chromosomes), there are undoubtedly potential multiple recombinant conformations in the *B. schreberi* mitogenome, including the possible split of chromosome 1 (Fig. 6). Various potential subgenomic forms or isomers of mitogenomes have been discovered in many plants, but high coverage of long-read data is required to detect these low-frequency genomic recombination events. We designed four pairs of primers in our experiment, through PCR experiments, successfully validating the potential structure of chromosome 1 mediated by repetitive sequences. The six circular molecules reported in this paper can represent the main existing forms of the *B. schreberi* mitogenome.

## Sequence migration and gene dialogue in organelles

Horizontal gene transfer (HGT) is a common phenomenon in prokaryotes, but it is rare in eukaryotes. The origin of terrestrial plants is believed to be associated with numerous HGT events from soil bacteria to their aquatic ancestors, laying a genetic foundation for terrestrial plants [25]. In flowering plants, there is a significant interaction of genetic elements between the two cellular organelle genomes, predominantly evidenced by the incorporation of chloroplast DNA into the mitochondrial genome. Up to this point, the converse phenomenon, where mitochondrial DNA integrates into the chloroplast genome, has been uniquely observed in *Anacardium occidentale* [69]. Additionally, there is evidence of HGT between organellar and nuclear genomes. Previously, HGT events in parasitic plants have been widely discussed. For example, some chloroplast genes in the mitogenome of the *Aphyllon epigalium* were transferred from its host's chloroplast genome [70]; the plastid-origin *rpl32* gene was reported to be transferred to the nuclear genome in the subfamily Thalictroideae [71]; the *atpI* gene found in the mitogenome of *Aeginetia indica* is believed to have originated from the chloroplast genome of another angiosperm [72]; In the genus *Geranium*, we observed multiple instances of horizontal gene transfers involving ribosomal proteins. However, studies suggest that these transferred genes from the plastid might be non-functional in the mitochondria, potentially becoming pseudogenes during mitogenome evolution [72]. Because we lack the nuclear genome data for *B. schreberi*, this study only analyzes the sequence transfer between the two organellar genomes of *B. schreberi*. As previously analysed, the *B. schreberi* mitogenome have absorbed a large number of DNA fragments from chloroplasts, including partial sequences of chloroplast PCGs. However, these fragments are mostly incomplete or contain internal stop codons, rendering them biologically non-functional in the mitogenome. Additionally, ten tRNA genes in the mitochondrial genome (*trnC-GCA, trnM-CAU, trnI-CAU, trnQ-UUG, trnN-GUU, trnT-GGU, trnW-CCA, trnA-UGC, trnI-GAU,* and *trnV-GAC*) were identified as having sequences identical to those in the chloroplast. These may be horizontally transferred, but further research is needed to confirm this. Also, *Wolffia arrhiza* mitochondria have their own *trnN-GUU* and *trnQ-UUG* genes, but lack *trnA-UAG* and *trnV-GAC*, which are also missing in the closely related *N. colorata*. Unlike the stable 22 tRNA genes in animal mitogenomes, plant mitogenomes often exhibit varying degrees of tRNA loss, relying largely on extramitochondrial tRNAs for amino acid transfer. *B. schreberi* mitogenome possesses 18 unique tRNAs; nevertheless, it still lacks tRNAs for isoleucine, leucine, and serine, which might largely depend on the corresponding nuclear-encoded tRNAs entering the mitochondrial matrix to compensate [73]. In summary, the chloroplast genome of *B. schreberi* provides a rich source of exogenous sequences for its mitogenome, even involving some gene transfers, contributing to the variability of the *B. schreberi* mitogenome and increasing the diversity of mitochondrial DNA sequence sources. Further research is needed to determine whether these genes are still functional.

### Supplementary Information

**Supplementary Material 1.**

**Supplementary Material 2.**

## Data Availability

The raw sequencing data for the Illumina and Nanopore platforms and the mitogenome sequences has been deposited in NCBI (https://www.ncbi.nlm.nih.gov/) with accession numbers: MZ919331.1 to MZ919336.1. The sample has been deposited in the herbarium of Southwest University, Chongqing, China, with voucher number: 20210425CC-1.
