# Supplementary material for: Elucidating the multichromosomal structure within the Brasenia schreberi mitochondrial genome through assembly and analysis
Source: BMC Genomics. 2024 Apr 29;25:422. doi: 10.1186/s12864-024-10331-0 (PMC11059650; doi:10.1186/s12864-024-10331-0)
Supplement: Supplementary file 1 — Supplementary Material 1. [file 12864_2024_10331_MOESM1_ESM.docx]

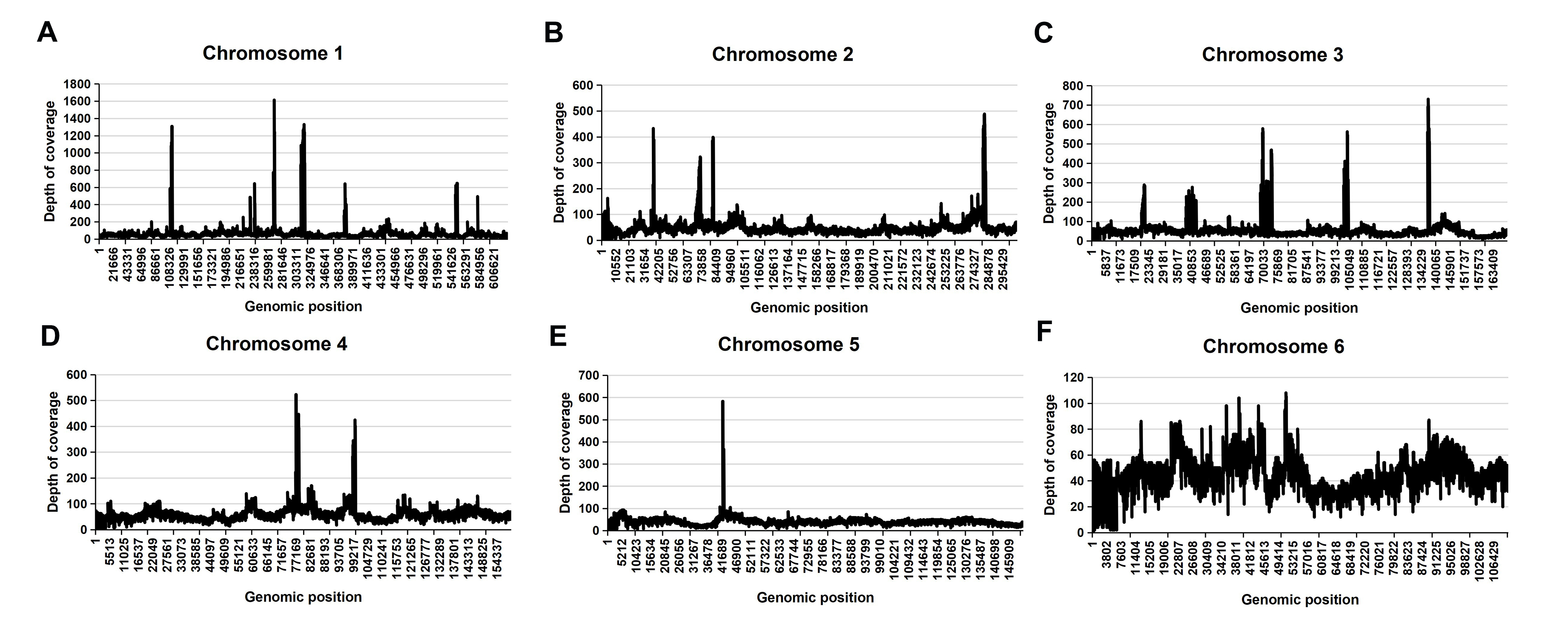


**Figure S1.** Sequencing depth of coverage based on Illumina short-reads.

**A-F** represent the depth of coverage from Chromosome1 to Chromosome6, respectively. The abscissa indicates the location of the contig, and the ordinate indicates the sequencing depth. The average depth of the six contigs was about ~80×.


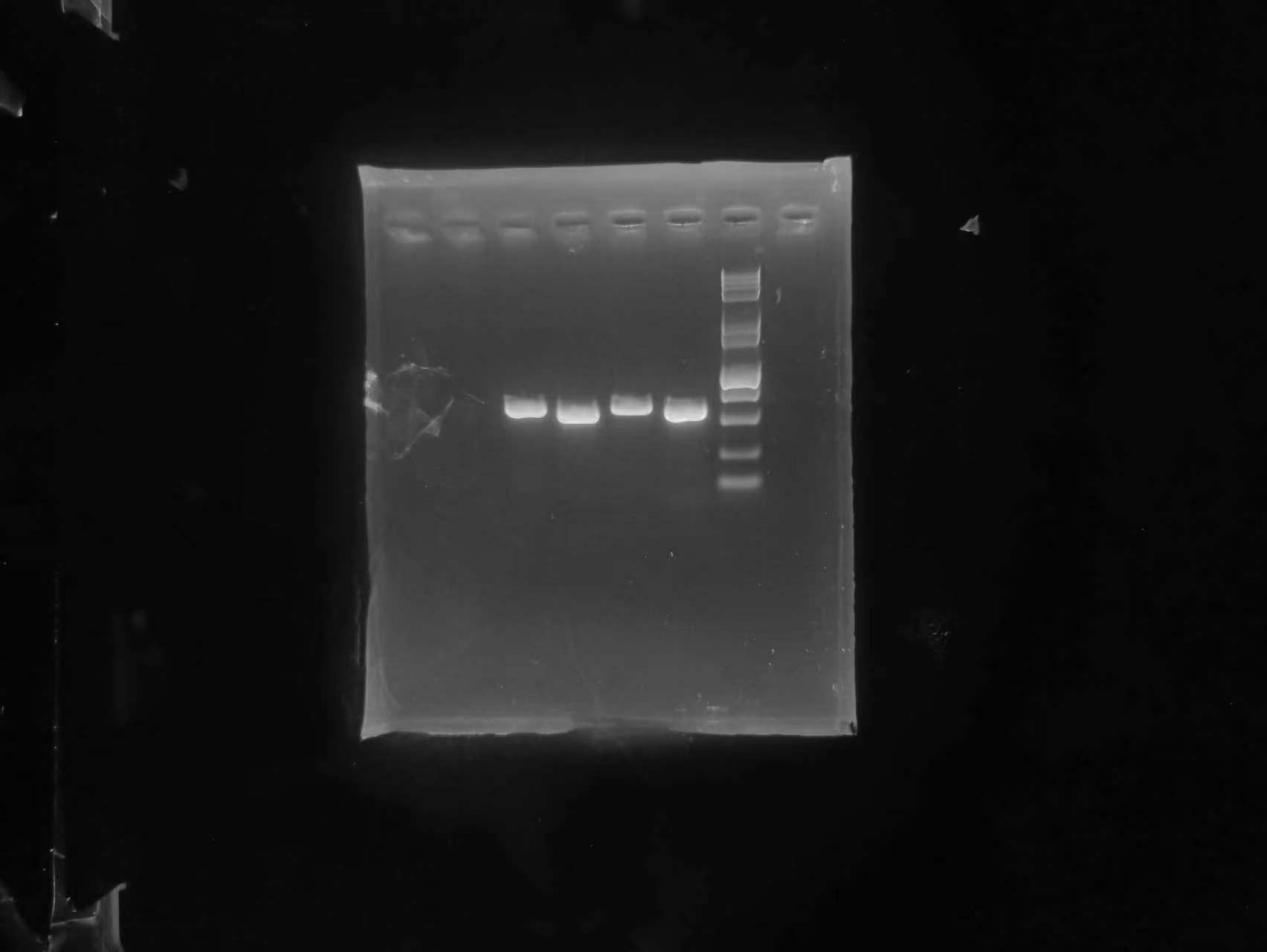


**Figure S2.** The original uncut electropherogram.


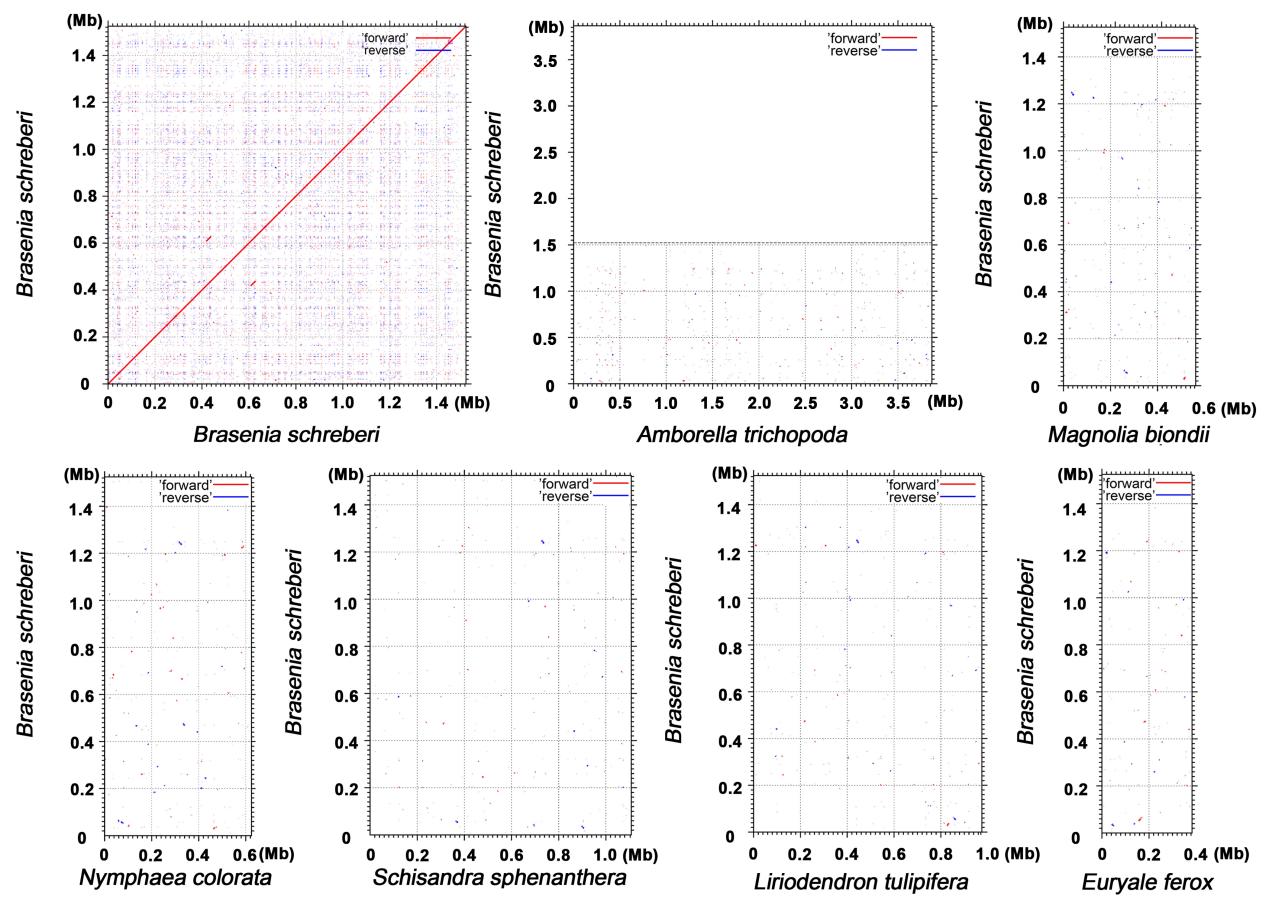


**Figure S3.** The self-dotplot of the mitogenome of *B. schreberi* and the dotplot of *B. schreberi* and its related species.
